# Digital Reconstitution of Road Traffic Accidents: A Flexible Methodology Relying on UAV Surveying and Complementary Strategies to Support Multiple Scenarios

**DOI:** 10.3390/ijerph17061868

**Published:** 2020-03-13

**Authors:** Luís Pádua, José Sousa, Jakub Vanko, Jonáš Hruška, Telmo Adão, Emanuel Peres, António Sousa, Joaquim J. Sousa

**Affiliations:** 1 Engineering Department, School of Science and Technology, University of Trás-os-Montes e Alto Douro, 5000-801 Vila Real, Portugal; jmsousa@utad.pt (J.S.); vanko.jakub@gmail.com (J.V.); jonash@utad.pt (J.H.); telmoadao@utad.pt (T.A.); eperes@utad.pt (E.P.); amrs@utad.pt (A.S.); jjsousa@utad.pt (J.J.S.); 2 Centre for Robotics in Industry and Intelligent Systems (CRIIS), INESC Technology and Science (INESC-TEC), 4200-465 Porto, Portugal

**Keywords:** road traffic accidents, reconstitution, unmanned aerial systems, photogrammetry

## Abstract

The reconstitution of road traffic accidents scenes is a contemporary and important issue, addressed both by private and public entities in different countries around the world. However, the task of collecting data on site is not generally focused on with the same orientation and relevance. Addressing this type of accident scenario requires a balance between two fundamental yet competing concerns: (1) information collecting, which is a thorough and lengthy process and (2) the need to allow traffic to flow again as quickly as possible. This technical note proposes a novel methodology that aims to support road traffic authorities/professionals in activities involving the collection of data/evidences of motor vehicle collision scenarios by exploring the potential of using low-cost, small-sized and light-weight unmanned aerial vehicles (UAV). A high number of experimental tests and evaluations were conducted in various working conditions and in cooperation with the Portuguese law enforcement authorities responsible for investigating road traffic accidents. The tests allowed for concluding that the proposed method gathers all the conditions to be adopted as a near future approach for reconstituting road traffic accidents and proved to be: faster, more rigorous and safer than the current manual methodologies used not only in Portugal but also in many countries worldwide.

## 1. Introduction

Regardless of the many road safety programs already implemented, undergoing or foreseen—e.g., the European Union (EU) is discussing their 5th Road Safety Action Programme for the 2020–2030 decade [1], intended to have a 50% reduction on both deaths and serious injuries between 2020 and 2030—related rates recorded worldwide are still significant. Indeed, the World Health Organisation (WHO) estimates that, annually, road traffic accidents kill around 1.25 million people and injure about 50 million more across the globe. In fact, in 2012, road traffic accidents were the leading cause of death between ages 15 and 29 years old [2]. Furthermore, the International Transport Forum (ITF), who gathers information from 40 countries in its 2017 Road Safety Annual Report [3], corroborates the aforementioned numbers related with casualties.

Besides the social consequences, road traffic accidents also imply considerable economic impacts. Wijnen and Stipdonk [4] estimated the economic impacts as a percentage of countries gross domestic product (GDP): an average of 2.7% GDP to those they regarded as high-income countries and 2.2% GDP to low and middle- income countries. Examples of road traffic accidents economic costs in some countries were presented by the ITF [3]. This enormous economic impact weights down on families, insurance companies (e.g., damages), healthcare systems (both public and private), companies (e.g., production loss) and society as a whole [2,4].

Road safety is usually related with human behaviour, physical infrastructures and vehicles [5,6] (detailed information about road safety factors can be found in Rune and Vaa [7]). Countless media campaigns are made worldwide each year to raise awareness and try to reduce on-road risk behaviours. WHO has even released a mass media campaigns toolkit to help low- and middle-income countries develop their own initiatives [8]. Physical infrastructures design and planning, as well as vehicles active and passive safety measures or regular condition checking aim at giving a contribution to improve road safety [9,10].

When all else fails and road traffic accidents happen, it is crucial to carry out a crash scene reconstitution with the utmost rigor and objectivity, to determine the causes underlying the event. This information is the basis in which all of the accidents subsequent stages lean on. Whilst investigation, insurances, legal aspects and property damages are stages that directly depend on crash-site gathered information. This information is used to determine the causes of the event which can potentially have a valuable contribution in the aftermath, namely in the reduction of road traffic accidents. Indeed, an accurate crash scene reconstitution may allow the identification of design flaws in physical infrastructures design flaws and/or risk behaviours that support their mitigation through physical interventions or by raising awareness.

Therefore, data collection at the crash site takes on a decisive importance [11]. Police forces are usually the first to arrive to a road traffic accident site. Ordinarily, their mission is first of all to assure victims (if any) proper medical assistance and then to secure the crash scene, preserving both property and evidences alike, followed by the traffic re-routing procedures. Afterwards, the investigation procedure starts by identifying and secure evidence, drawing sketches (e.g., vehicles positions and paths, visible traffic signs), take note of on-site conditions (e.g., visibility), take measures and sometimes, acquire photographs for future memory [12]. Besides the foremost objective to preserve life, restoring normal traffic flow to the affected roads is a prime directive, avoiding yet more social and economic impacts.

In addition to the difficulties connected with the need of collecting a high amount of information on site, sometimes measurements taken from specific references cannot be met. For example, in what regards distances from the wheels of the vehicles, these should be measured from their centre and not from the edge of the tire. Moreover, this must be done from the same location, to the reference point. Under adverse weather conditions, there is also the risk of some evidences becoming less visible or even vanish. Furthermore, if pedestrians are involved in the accident, the information to be gathered increases considerably.

Thus, data collection on site should be a simple, precise and swift procedure. However, currently it is mostly a manual procedure done by police forces worldwide. Although there might be slight differences from country to country, data collection methods used when road traffic accidents occur are very similar. They are based mainly in measurements done manually, using triangulations and parallel lines to produce the crash scene sketch: as such, both rigor and precision of a road traffic accident scene sketch depend mostly on the professionalism of the police forces. These difficulties can be overcome using small-sized, lightweight and cost-effective multi-rotor unmanned aerial vehicles (UAVs), capable of operating in fully automatic mode or by manual control, which constitute the technological core of Unmanned Aerial Systems (UAS)-based photogrammetric methodology proposed for road traffic accidents reconstitution. More than providing scientific documentation, this technical note intends to supply road traffic authorities and professionals with a set of procedures compiled in a UAV-based methodology that aims to achieve accurate, efficient and cost-effective digital reconstruction and recording of motor vehicle collision events.

## 2. Background

Recent photogrammetry methods were boosted by solid and fast pace developments carried out in digital image processing and computer vision. Some feature extraction algorithms—for example, scale invariant feature transform (SIFT) [13] and structure from motion (SfM) analysis [14].Due to its capability of generating wide and detailed digital data sets faithfully portraying real-world sites, photogrammetry has been gradually adopted in many areas of application [12]. One of them is the reconstruction and analysis of road traffic accidents in several projects and countries. A report provided by Arnold et al. [15] explores alternatives to the laborious and time-consuming traditional road traffic accident surveying methods. Close-range photogrammetry is highlighted. In spite of the resistance that is still noticed in adopting advanced techniques, many law enforcement entities around the globe admit the benefits of photogrammetry in both ease of use and time-saving factors, which has been leading to progressive changes in road traffic accidents surveying operations [16]. Zuraulis et al. [17] showed the relevance of photogrammetric systems for road traffic accident investigation, specifically by using tire yaw marks as evidences. This study suggests that the speed calculation methodology depends on tire curvature conditions, with lower errors for shortest chord lengths.

Photogrammetric methods demonstrated to be useful for substantial accuracy improvement in speed determination: around 3% error was obtained when using curvatures shortest chord lengths, which is an acceptable value for forensic practices. However, some disadvantages regarding close-range photogrammetry have been noted [18].

With aerial photogrammetry, namely using Unmanned Aerial Systems (UAS)—that combine a UAV, sensors as payload and a ground station, which has software for flight management and planning [19]—both complexity and laborious road traffic accidents surveying operations dropped significantly, comparatively to the previously referred approach, relying on multi-positional photographs. This technological advancement turned UAV-based photogrammetry into a desirable precise and efficient tool for business-to-business companies and law enforcement entities dealing with road traffic accidents which has been also drawing the attention of researchers. Monadrone [20] used a UAV to acquire images from a simulated accident scene. The Royal Canadian Mounted Police and Pix4D company cooperated with the goal of comparing terrain surveying methods for road traffic accidents investigation—including laser 3D scanning—with UAV-based image acquisition [21]. The UAV-based photogrammetry reliability over traditional methods and its affordability comparatively to other precision instruments—e.g., laser scanners—were highlighted, along with other aspects such as view angles covering. A solution proposed by the Analist Group [22] resorts to terrain measurements and a UAV flight to obtain data for mosaicking and 3D model processing, using Pix4Dmapper Pro, both portraying different perspectives of a staged road traffic accident. Subsequently, measurements can be done in a computer aided design (CAD) software, that also supports simulating events sequences. Greg Gravesen, a specialist in road traffic accidents reconstitution who uses a fixed-wing UAV to create detailed representations from orthophoto mosaics are the base for the various simulations, as well as 3D meshes to create graphical hypothesis [23]. Crash scenes are then reconstructed and simulated using a dedicated software.

Some research has also been done in this area by Su et al. [24] which proposes a mapping system that applies UAVs to rapidly set up a scene representation for road traffic accidents investigation, as an alternative to the traditional hand-drawn sketches. Diaz-Vilarino et al. [25] identified a non-solved question on runoff evaluation. They carried out a study to determine the influence of data quality obtained using UAV-based photogrammetry in the calculation of a mountain road run off, when compared to LiDAR data. A method to check the limits of using point clouds obtained from UAV photogrammetry relying on the D8 (flow direction) algorithm was proposed. Data acquisition of road traffic accidents comparing traditional methods with advanced techniques, namely using total stations and photographic documentation that can also involve UAV missions, was carried out by Stáňa et al. [26]. Results analysing real occurrences as case studies pointed out both faster processing and quicker road clearance, as well as (almost) error-free measurements, when applying advanced techniques. A Sheriff of Colorado State (USA) testified for a report [27], sharing the same thoughts. X. Liu et al. [28] proposed a framework method that integrates UAVs to achieve 2D/3D accident scene reconstructions, highlighting the enhanced capabilities of those platforms to cover more angles without the need to interrupt traffic flow. The results seem to attest this kind of approaches to digitally record casualties in road traffic accidents. Concerned with mosaicking production, Y. Liu et al. [29] developed a method that includes image registration and fusion, as well as intelligent distance measurement based in Euclidian distances, a case study was used to demonstrate the whole pipeline. In Stevens Jr et al. [30], UAS were used for real-time monitoring of secondary crashes, including alternate routes monitoring. The report highlights UAS capabilities to deliver imagery and sensory data on-the-fly, their safety during missions, as well as payload mobility. Coarse 3D reconstructions were also presented to demonstrate mapping capabilities for future access, but quality measurements were not carried out. Mekker [31] evaluated some consumer grade UAS for photogrammetric traffic crash scene documentation.

## 3. Road Traffic Accidents Reconstitution

### 3.1. Current Approach

The current procedures commonly used by police forces in road traffic accidents reconstitution relies on: a measuring tape and/or measuring wheel; a notepad, to draft the sketch; a pencil; chalk, to mark the pavement; and a camera for photographic documentation. Some constraints regarding the current data collection method are identified: (i) it involves a significant amount of time spent at the crash site by police officers that could otherwise be engaged in different tasks; (ii) it causes a disruption in regular traffic flow, with consequent economic and social impacts; (iii) it can potentially lead to losing valuable data, due to the lack of detail level in the sketch; and (iv) it exposes both police officers and civilians to unnecessary risks during the data collection process.

**Figure 1:**
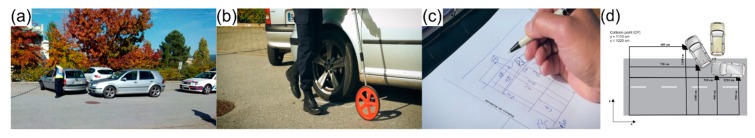
Current data collecting method applied by the Portuguese Public Safety Police (PSP) of Vila Real (Portugal) in a simulated road traffic accident scene: (**a**) staged traffic accident scene; (**b**) police officer using a measuring wheel to acquire data; (**c**) in situ sketch example; and (**d**) traffic accident scene sketch produced for clarification purposes by using an online platform.

Figure 1 depicts the current road traffic accidents data collection method applied to a simulated accident scenario. For this purpose, a simulation was conducted at the University of Trás-os-Montes e Alto Douro (UTAD), Vila Real, Portugal, with the cooperation of the Portuguese Public Safety Police (PSP) force from Vila Real, to ensure that the data collection process proceeded as if in a real road traffic accident scenario. Measurements done by PSP officers are in a local cartesian coordinate system: in this simulation, a lamp pole was used as the reference (x = 0.0 m; y = 0.0 m) and all measurements were referenced in relation to it. Moreover, a handmade sketch of the road traffic accident scenario was drawn into the notepad in situ (Figure 1c). For clarification purposes, the simulated road traffic accident was represented through a sketch drawn using an online platform (AccidentSketch.com) and is presented in Figure 1d. Besides measurements, some photographs are also usually required for documentation purposes. In this simple road traffic accident scenario, PSP took about 30 min to complete the data collection process. The handmade road traffic accident sketch (Figure 1c) was later used in the office to generate a digital version using a proprietary software. It was then included in the official accident report. This process took a few more hours of the police officers’ time, which could otherwise be used in more meaningful police work.

### 3.2. Proposed Approach

Considering a set of requirements aligned with the cooperation of Portuguese police forces, some preliminary technical decisions regarding the selection of hardware (UAV, camera and base station) and software (UAV flight planner/manager and photogrammetric tools) were specified. Relying on such requirements and specifications, the outline of a novel methodology to digitally reconstruct and record road traffic accidents was proposed to complement or, eventually, supplant traditional procedures.

#### 3.2.1. Requirements Definition

Defining a set of requirements to a methodology that, from the outset, is intended not only to demonstrate its feasibility but also to effectively be deployed and employed in real road traffic accidents scenarios, can be a demanding task. As such, cooperation was sought from the Portuguese National Road Safety Authority (ANSR)—whose mission is the “planning and coordination at national level to support the Government policy on road safety, as well as the application of the law on road safety”—and both police forces that deal with road traffic accidents in Portugal: PSP and National Republican Guard (GNR).

This cooperation resulted in the following set of requirements to which a UAS, to be used in road traffic accidents scenarios by police forces, must comply to be deemed suitable: (1) balanced relation between cost, reliability and precision; (2) compact, easy to manage, to transport and to assemble; (3) able to operate in both urban and nonurban contexts; (4) support both autonomous and manual flight modes; (5) high manoeuvrability and adaptability both in autonomous and in manual flight modes; (6) enable flights parameterisation to support a multiplicity of accident scenarios, namely regarding different types of trajectories, camera angle and images overlap; (7) autonomy to acquire data in typical road traffic accidents scenarios, using only one battery (assuming that most of road traffic accidents involves between two to three vehicles and that a common rotary-wing UAV can fly up to twenty minutes with only one battery, one flight is enough to cover the large majority of occurrences. Regarding multiple vehicle collisions in motorways, crash scenarios with highly dispersed debris or even involving explosions, the proposed methodology can be also addressed, but they may imply the use of more than one battery to fully cover the crash scenario); (8) integration of an accurate Global Navigation Satellite System (GNSS) receiver for positioning and imagery georeferencing; (9) support for RGB sensors that acquire high quality and high-resolution images up to 120 m altitude, which is the legal limit allowed for UAVs, according to European regulations (UAV flights are forbidden in some urban contexts, but as the proposed methodology will be applied by police forces, these situations must be foreseen in the legislation); and (10) allow for real-time access to the RGB sensor, through the ground control station, enabling the on-scene police officers to monitor and manage the data acquisition process and make an on-the-fly assessment about data being acquired.

Regarding post-flight data processing, more specifically the photogrammetric processing, another set of specifications was created for selecting the photogrammetric tools: (1) support for photos positioning and alignment, aiming the global or local data georeferencing and feature extraction; (2) capability for data scaling operations; (3) intuitive and easy-to-use software; (4) support for accurate dense point clouds; (5) support interactive highly accurate measurements and positional control; and (6) outputs such as orthorectified GIS-based products and virtual models.

#### 3.2.2. Hardware and Software Specification

Considering the set of requirements defined for UASs, the process of selecting the equipment deemed suitable for the proposed methodology and for this study purpose started off by addressing aspects such as packaging, transportation and ease of handling. Among the available types, small-sized rotary-wing UAVs superseded fixed-wing UAVs due to their ability to perform vertical take-off and landing (VTOL) and to maintain a stationary position during flight. The authors experience in working and operating with different UAVs also weighed in when selecting the two off-the-shelf UAV models for this study: DJI Phantom 4 and its predecessor, DJI Phantom 3 Standard (DJI TECHNOLOGY CO., LTD, Shenzhen, China).

For mission planning and execution, two software tools were used: DJI GO (DJI TECHNOLOGY CO., LTD, Shenzhen, China) and Pix4Dcapture (Pix4D SA, Lausanne, Switzerland). While the former stands as a valid option to test UAV configurations, the latter is suitable for preparing and executing mission flights that can be carried out manually or autonomously—in simple or double grid mode—and also for selecting which area to cover, the flight height and imagery overlap.

To complement UAV-based imagery, ground-perspective images can be collected by using ground cameras, namely smartphones belonging to a widely spread class of cost-effective consumer-grade technological devices, with built-in GPS capabilities and equipped with fairly acceptable quality cameras (12 MP). With these, police officers are able to deal with less accessible areas in the crash scene that cannot be tackled with—only or at all—using UAVs, due to the presence of obstacles (e.g., dense vegetation, poles, cables or buildings).

After acquiring data from road traffic accidents scenarios, a photogrammetric processing stage occurs. As such, notwithstanding several fully compliant solutions (some of them opensource and/or free to use), Pix4Dmapper Pro (Pix4D SA, Lausanne, Switzerland) was selected for this study. The decisive factors for this choice were the research team significant experience in using this software and an available license.

The resulting digital products from this pipeline represent a realistic and complete set of elements that can help to reconstitute road traffic accidents scenarios.

#### 3.2.3. Environmental Conditions Variability

UAV-based photogrammetric approaches for road traffic accidents reconstitution are usually equipped with one RGB camera to acquire images from the scene and GNSS, which besides georeferencing each image, enables autonomous flights throughout pre-established set of waypoints (i.e., mission plans). As such, human intervention can be minimised during the data acquisition process, thus reducing the potential for errors. However, some context conditions might significantly influence this process, namely (1) the presence of unmovable obstacles that partially or completely prevent UAVs access to the crash scenario; and (2) adverse light conditions, often found with some environmental phenomena and/or at night. Acquired images are then processed by using a photogrammetric software to accurately reconstitute the traffic accident scenario.

#### 3.2.4. Methodology Overview

When a road traffic accident happens and assuming that police forces are called to the scene, the proposed methodology starts of by having officers evaluate the crash scenario (e.g., area of interest, number of vehicles involved, dispersion of potential elements of interest) and the context conditions (e.g., environmental and light conditions and the presence of obstacles). Indeed, four road traffic accident scenarios are foreseen, each with its corresponding imagery acquisition process, as summarised in Table 1.

During the imagery acquisition process, a small number of distances between reference points can be measured to ascertain the road traffic accident scenario reconstitution quality and also for calibration purposes: bars with well-known dimensions can be used (placing them in the area of interest in concurrent directions), measuring tape (or wheel) or by employing a GNSS receiver. Although it is worth mentioning that if the objective is to reconstitute a road traffic accident scenario circumscribed to a relatively small area (a few square meters) by using a UAV equipped with a precise GNSS receiver (with errors around a few centimetres), then the distance measurement between reference points may be unnecessary, due to photogrammetric software capabilities in estimating 3D positions with acceptable accuracy.

**Figure 2:**
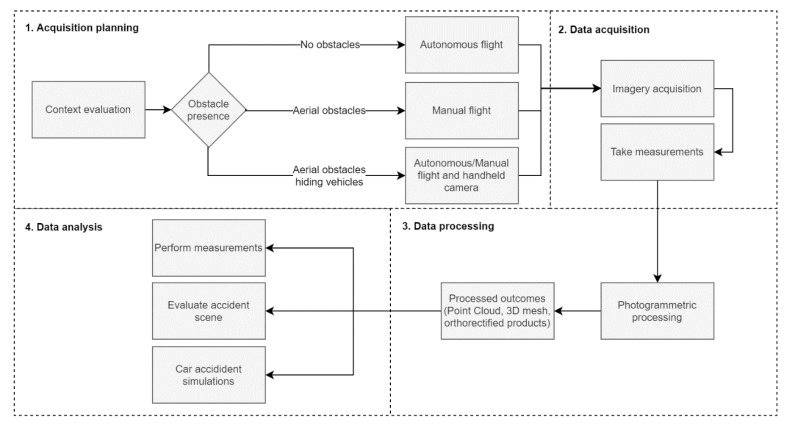
Unmanned aerial vehicle (UAV)-based road traffic accidents reconstitution methodology functional diagram.

Lastly, the acquired data concerning the road traffic accident scenario must be processed using a photogrammetric software to obtain an orthophoto mosaic and a virtual model reconstruction, therefore digitally documenting the road traffic accident. The crash scenario reconstitution is represented by a georeferenced 3D model or point cloud, in which precise measurements can be taken, edited or complemented at any point in time, through the use of specific software capable of dealing with Geographic Information Systems (GIS) data and/or Computer Aided Design (CAD) models. Figure 2 presents the proposed methodology functional diagram.

## 4. Experimental Tests: Fine-Tuning Parameterisation for the Proposed Methodology

### 4.1. Experimental Scenario

A set of preliminary tests, considering the most suitable context conditions (clear sky, obstacles-free wide-open area), were performed with various heights and distinct camera angles in order to assess the proposed methodology feasibility. These tests were also used to determine flight parameters default settings such as flight duration, most suitable number of acquired images, processing time, Ground Sample Distance (GSD) and measurements precision. More specifically two experimental tests sets were completed: one to optimise flights height and duration, as well as the resulting digital products overall quality, including resolution and 3D model detail; another to compare both precision and execution time of the current data collection method and the proposed methodology, within a road traffic accident scenario context. As a result, near-optimal parameter combinations were determined for future reference.

### 4.2. Flight Height and Camera Angle

Both flight height and camera angle are important parameters inasmuch as they influence orthorectified and virtual model outcomes obtained through photogrammetric processing of imagery acquired in a given area of interest. Regarding camera angle, nadiral images would allow for obtaining the best orthorectified mosaics. However, it is a less favourable situation for obtaining 3D models due to a poorer perspective coverage. With regard to flight height, it stands to reason that lower flight heights allow for a higher spatial resolution. This can be important, to detect vehicles damage and existent debris. Nevertheless, in road traffic accident scenarios, namely those in urban contexts, lower heights can induce more operational constraints and hamper the detection of similar features in extreme situations, with a negative impact on the photogrammetric processing. Experimental tests were conducted to find the best possible compromise between these two parameters, aiming to obtain the most suitable outcomes from the photogrammetric processing.

**Figure 3:**
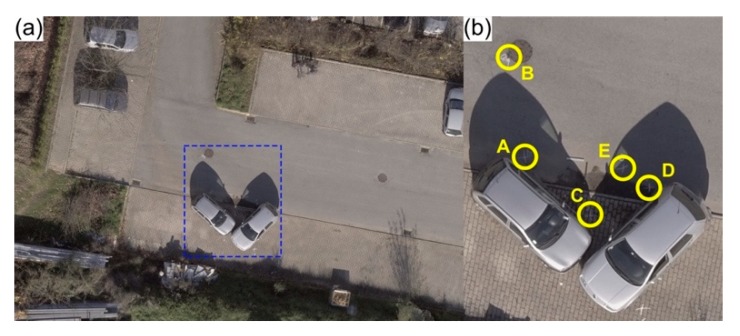
Simulated scenario to determine the best possible compromise between flight parameters for traffic accidents reconstitution (**a**). The area considered for measurements was of 67 m^2^, which corresponds to the blue dashed polygon (**b**), five measuring points are highlighted (A—E).

To test and assess combinations between these two parameters, several flight missions were carried out under ideal circumstances (clear sky sunny days with weak or non-existent wind, within an obstacle-free wide-open area), establishing accidents scenarios in controlled conditions, as presented in Figure 3.

Flights height varied between 5 m and 100 m, with 5 m increments. Flight missions were carried out manually for both the 5 m and 10 m heights. The purpose was to simulate a situation where there were eventual obstacles present, while maintaining a clear line-of-sight with the UAV. The remaining flights were all done in fully autonomous mode, using the same mission plan in Pix4Dcapture. The influence of different perspectives was evaluated by carrying out both simple and double grid flight missions. With regard to camera angle, it stands to reason that it must be sharper in lower height flights and wider (until nadiral position) in higher height flights, to be able to capture accident scene elements. It varied from 30° to 60° in flights where the height was equal to or lesser than 10 m and from 60° to 90° for the remaining flights. To assess both positional rigor and model generated scaling, two perpendicularly arranged metal bars, with a known length (1 m), were placed within the simulated road traffic accident scenario. Five points—from A to E—were marked in the pavement using chalk, for validation purposes (Figure 3b).

Table 2 presents data regarding the most representative combinations of the following parameters: type of flight mission—manual or autonomous (simple/double grid), flight height, camera angle, number of acquired images, flight mission duration, GSD and photogrammetric processing duration. As the flight height decreased, flight duration increased, along with the number of acquired images and the GSD. Furthermore, the number of acquired images demanded more computational resources during the automatic photogrammetric processing (therefore excluding manual operations, such as scale constraints handling and measurements determination), which increased its duration.

Tape measurements made between the five points (from A to E) marked in the pavement of the road traffic accident scenario (Figure 3b) were used as reference to compare to those extracted from the photogrammetric-based virtual model, generated by Pix4Dmapper Pro. This allowed for both assessing and validating the proposed method accuracy. Table 3 presents the most representative results obtained from the different flight missions. Overall, lower flight heights, and therefore higher spatial resolutions, enabled more accurate measurements. The most accurate result was achieved by a double grid flight, carried out at 15 m height with the camera angle set to 65°.

### 4.3. Precision: Can a UAV-Based Photogrammetric Approach Be Comparable?

**Figure 4:**
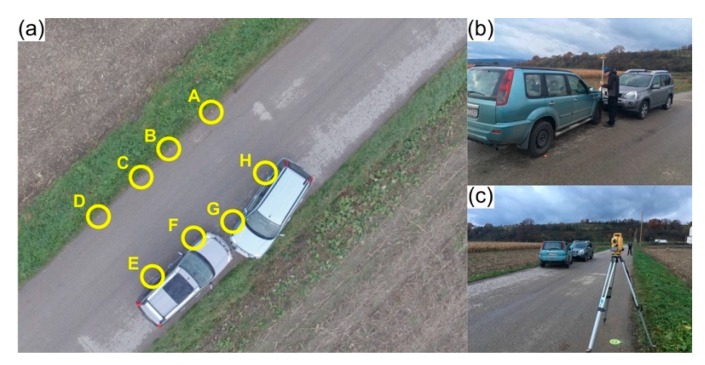
Assessment of different measuring methods in a car accident scenario: (**a**) aerial view of the road traffic accident scenario, with the eight points (A–H) considered for comparing the measuring methods; measuring distances using (**b**) the GNSS receiver; and (**c**) the total station.

Another road traffic accident was simulated to assess the precision of photogrammetric-based outcomes, by comparison, with a GNSS receiver and a total station. Validation was achieved using measurements taken with a measuring tape as ground-truth. Figure 4a presents the 30 × 30 m accident scene, where eight points were considered (A, B, C, D, E, F, G and H) and nine distances were measured. Furthermore, the time required to apply each measuring procedure—proposed method, total station, GNSS receiver and tape—was also measured.

Current procedures that are commonly used in road traffic accidents’ reconstitution by police forces are based on a measuring tape (or wheel). As such, measurements acquired using this method were considered as reference or ground-truth. The nine distances (AB, BC, CD, EF, GH, AH, BG, CF and DE) were measured in approximately three minutes. Regarding GNSS, measurements were taken in approximately eight minutes with a Topcon HiPer II GNSS receiver (Topcon Corporation, Tokyo, Japan), while ensuring a positional precision between 2 and 4 cm, in kinematic mode. As for the total station, a couple of additional points with known coordinates were considered for orientation purposes, besides the existing eight. Using a Topcon GPT-3005N equipment, the entire process took about nine minutes and 30 s (equipment configuration and data acquisition). The proposed UAV-based method was applied based on knowledge presented in the previous section and planned using Pix4Dcapture: an obstacle-free accident scene allowed for an autonomous flight at 15 m height, with a 65° camera angle and 75% images overlap. A total of 42 images were acquired in approximately 3 min and 50 s. Images were processed in approximately 2 min using Pix4Dmapper Pro running on a computer equipped with a 2.6 GHz Intel i7-4720HQ CPU (Intel Corporation, California, United States of America), 16 GB memory (1600 MHz) and a NVidia GeForce GTX 970M (Nvidia Corporation, California, United States of America) GPU. The resulting mosaic had a 0.55 GSD. Table 4 presents the measurements acquired by each addressed method.

GNSS presented the most accentuated deviations regarding ground-truth. When compared with the (high-precision) total station, measurements taken using the proposed UAV-based method presented a quite similar accuracy, but with better time regarding operational performance. Moreover, operating a UAV involves less effort due to the autonomous flight capabilities. Another advantage when using the proposed approach is that it provides more data for road traffic accidents reconstitution—through acquired aerial imagery conversion into orthophoto mosaics, dense point clouds and textured virtual models—that can be achieved by resorting to user-friendly photogrammetric software tools.

## 5. Experimental Tests: Simulated Scenarios

The proposed methodology proved to be a valid approach for the reconstitution of road traffic accidents scenarios occurring in obstacles-free wide-open areas, under clear sky sunny days. However, to be deployed and used by police forces under real scenarios, this methodology needed to be assessed in less favourable conditions. Obstacles that can commonly be found in urban and non-urban environments, such as aerial electrical cables, buildings, walls, vegetation and trees, were considered in simulated crash scenarios. Low light conditions or even night-time environments were also considered in order to fully evaluate the proposed methodology. Considering the findings documented in Section 4.2, all flights presented hereinafter had images overlap between 70% and 80%. All measurements of the tests performed in this section were taken using a measuring tape.

### 5.1. Scenario 1: Poles and Electrical/Communications Aerial Cables

**Figure 5:**
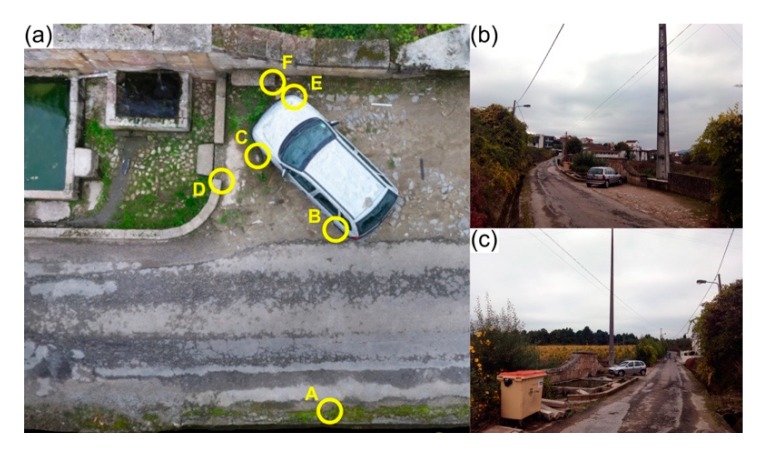
Road traffic accident simulation in a context with poles and aerial cables: (**a**) aerial view of the road traffic accident scenario, where six control points (A–F) were marked to compare results between the proposed approach and ground-truth measurements; (**b**) and (**c**) different perspectives of the accident scene, where the poles and aerial cables are visible.

The first scenario was simulated in an area with electrical and telecommunications poles, which are very common obstacles in both rural and urban environments. As can be observed in Figure 5, their presence does not favour UAV flights at lower flight heights using autonomous flight modes. Under these conditions, a manual flight mode—providing full flight control to the operator and thus the responsibility to avoid/bypass obstacles—seems to be the most reasonable option for acquiring data in a safe way for both police officers and equipment. In this simulated scenario the camera gimbal was manually controlled to adopt several orientations. The goal was to ensure a suitable overlap between images and to properly cover all the scene during on-the-fly data acquisition, towards a faithful and highly detailed 3D reconstitution. A total of 119 images were acquired in about four minutes. Figure 5 presents an aerial view of the simulated crash scenario, with six control points (A, B, C, D, E and F).

A subset of measurements—AB, CD and EF—for comparison with the resulting point cloud is recorded in Table 5. The maximum error is of 2 cm (0.44%) was observed in AB.

### 5.2. Scenario 2: Presence of Trees with Large Canopies

**Figure 6:**
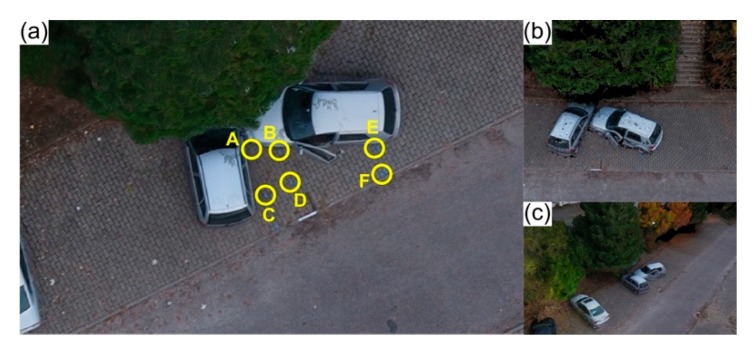
Road traffic accident simulation in which trees partially obstruct the involved vehicles. Aerial view of the scene and the six control points marked for measurement (A—F) (**a**); generated dense point cloud (**b**); and the 3D model resulting from applying the proposed methodology (**c**).

Another common situation is the existence of natural obstacles in a road traffic accident scenario. A second simulated scenario was set in such a way that large trees obstructed portions of the involved vehicles and other possible elements of interest. Figure 6 depicts both the road traffic accident scenario (Figure 6a) and the 3D representations from the photogrammetric processing (Figure 6b,c). In the latter it is clear that the crash scene portions obstructed by the trees canopy cannot be reconstituted by solely using aerial images. Regarding UAV imagery, 43 images were obtained with approximately 2 min double-grid autonomous flight, at 30 m height and with a 70° camera angle.

Table 6 presents a subset of results—AB, BD, CD, AC, EF—comparing the ground-truth measurements to the ones obtained from the resulting point cloud. All distances are very similar in both methods, with a maximum error of 1 cm occurring in all measurements.

However, a complete crash scene reconstitution was not possible, due to the obstruction caused by the trees canopy, which led to the aerial images being useless for that part of the scenario. As such, digital outcomes from the proposed methodology are somewhat incomplete, as can be seen in the 3D model (Figure 6c). To overcome this issue and as mentioned in Section 4.2, ground-based image acquisition can be integrated with UAV-based imagery, thus enabling a full and accurate crash scenario reconstitution when parts are obstructed and not visible in the aerial imagery.

### 5.3. Scenario 3: Presence of Dense Vegetation

**Figure 7:**
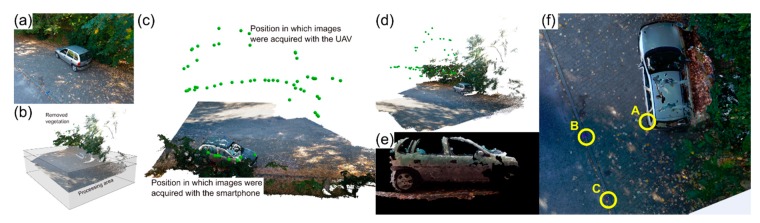
Road traffic accident simulation with one vehicle in a dense vegetation context: (**a**) vehicle aerial view; (**b**) point cloud vegetation filtering; (**c**) dense point cloud combining both ground and aerial imagery; (**d**) resulting UAV-based point cloud; (**e**) resulting 3D model of the vehicle side obstructed by vegetation, using ground-based imagery; and (**f**) part of the resulting orthophoto mosaic, highlighting in situ measuring points (A–C) used for precision assessment purposes.

This third simulated road traffic accident presents a scenario where a significant portion of a vehicle and possible other elements of interest are obstructed by dense vegetation (Figure 7a). This situation blocks the aerial view in part of the scene. Images acquired from the ground, using a smartphone equipped with a 13 MP camera, were thus combined with the aerial imagery. UAV-based imagery acquisition was carried out in a manual flight that took approximately 4 min and 30 s, at a variable height between 5 to 10 m and with a 70° camera angle, enabling a complete accident scene reconstitution.

Before the proposed methodology processing stage, the vegetation was removed by defining a sub-region of interest focused on the vehicle (Figure 7b). A total of 50 aerial images were acquired using the UAV, which enabled the computation of a dense point cloud (Figure 7d). As the dense vegetation prevented the reconstitution of one of the vehicles sides, to document that missing part, 17 images from the obstructed area were acquired using the smartphone camera—this process took approximately three minutes—and used to complement the aerial imagery (Figure 7e). Lastly, three measuring points (Figure 7f) were marked on the ground (A–C), to compare the reference measurements and the ones obtained from the resulting point cloud.

The comparison between ground-truth measurements (AB 209 cm and AC 308 cm) with the ones obtained from the final dense point cloud (AB 210 ± 1 cm and AC 309 ± 1 cm), showed, again, only 1 cm discrepancy between them, proving that both ground and aerial imagery can be combined to obtain a precise road traffic accident scenario reconstitution using the proposed methodology.

### 5.4. Scenario 4: Presence of Buildings

This fourth scenario simulates a road traffic accident in which at least one of the involved vehicles is fairly close to a building, thus having one of its sides obstructed to autonomous aerial imagery acquisition. The aim is to depict a common situation, especially in urban contexts. Regarding UAV imagery, 40 images were obtained with an approximately 4 min and 30 s autonomous flight, at 20 m height and with a 70° camera angle. Furthermore, a manual flight was also performed varying flight height during the mission, while maintaining the camera angle constant. This flight aimed to document the vehicle obstructed portion, thus complementing the data acquisition task.

**Figure 8:**
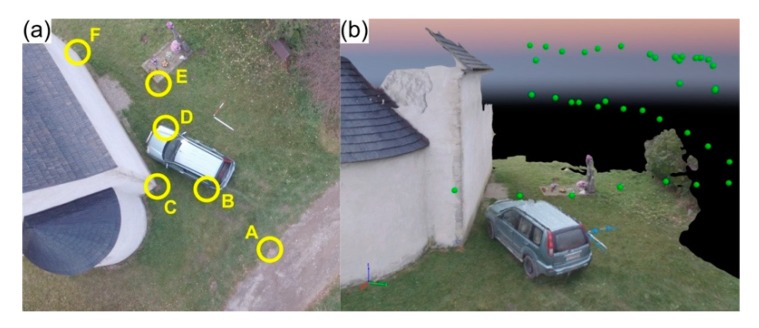
Road traffic accident simulated scenario near buildings: (**a**) aerial overview of the scene along with the six control points (A–F) marked for precision assessment; (**b**) 3D model reconstituting the crash scene from the photogrammetric processing of the aerial imagery, green dots represent the camera positions.

Figure 8a presents this simulated crash scenario and the six points (A–F) marked on the ground for precision assessment. Figure 8b presents the final 3D model obtained through photogrammetric processing of the UAV-based imagery acquired by both the manual and automatic flights.

Table 7 compares the ground-truth measurements with the ones taken from the dense point cloud. A 1 cm maximum potential error in DE demonstrates the feasibility of this approach, proving that imagery from both autonomous and manual flights may be combined to obtain a precise road traffic accident scenario reconstitution using the proposed methodology.

### 5.5. Scenario 5: Light Conditions

Besides optimised flight parameters, imagery quality is key to obtaining reliable and accurate reconstitutions of road traffic accident scenarios, using the proposed methodology. While physical obstructions to aerial imagery can be managed by using complementary acquisition equipment and techniques, light conditions can play a central role regarding imagery quality. Insufficient light conditions in a road traffic accident context require artificial light sources to apply the proposed methodology, as it is highly dependent on the imagery quality to deliver complete and accurate digital outcomes. A lighting kit for DJI Phantom 4 UAVs was selected (Lume Cube, Seattle, WA, USA). It was composed by two Light Emitting Diode (LED) lights—each a 3.81 cm cube, weighting in 28.35 g (battery included), supplying 750 LUX at 1 m, with a 60° beam angle and an adjustable position angle—and all the necessary parts to assemble it in the UAV. As for ground-based illumination, four Aputure Amaran AL-H198 LED lights (Aputure Imaging Industries Co. Ltd., Shenzhen, China) were selected. Each unit measures 23.4 × 9.1 × 14 cm (length × width × height), powered by battery (5.5V–9.5V DC), weighs 325 g, supplies 920 LUX at 1 m, with a 60° beam angle, including adjustable light intensity and adjustable colour temperature.

**Figure 9:**
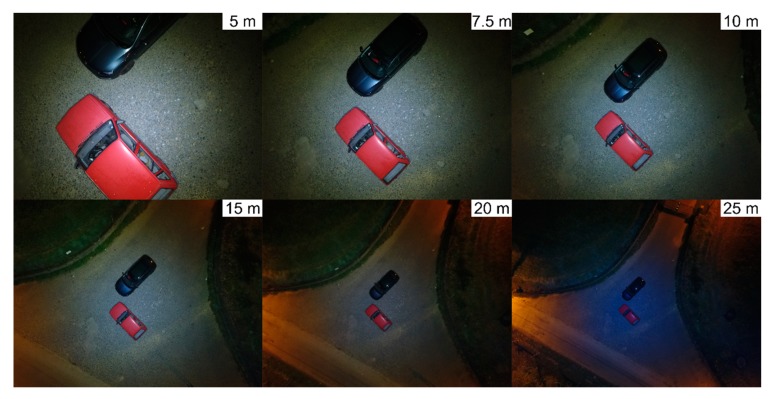
Aerial images acquired without public illumination support at different heights, with portable illumination coupled to the unmanned aerial vehicle (UAV).

The first simulated road traffic accident scenario under adverse illumination conditions was intended to visually assess acquired aerial imagery. The simulation took place in a suburban area, without direct light from nearby public illumination and involving two vehicles. Relying only on portable illumination coupled to the UAVs, images were taken in the same point at different heights (5 m, 7.5 m, 10 m, 15 m, 20 m, and 25 m) over the scene, as shown in Figure 9. An image was acquired in a manual flight, with the UAVs RGB camera in a nadiral position.

Portable illumination coupled to the UAVs is enough to document road traffic accidents scenarios with high visual quality, under adverse illumination conditions, to heights equal or inferior to 15 m. Flights over that reference height cannot be dismissed, mainly because obstacles may be present in the scene. However, images visual quality decreases as flights height increases (Figure 9).

**Figure 10:**
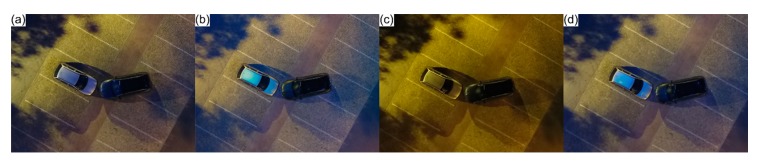
Orthophoto mosaics generated from aerial imagery acquired in an adverse light context: (**a)** and (**c**) only supported by public illumination; (**b**) and (**d**) public illumination combined with the portable one coupled to the UAV; (**a)** and (**b**) at 20 m height; and (**c**) and (**d**) at 30 m height.

**Figure 11:**
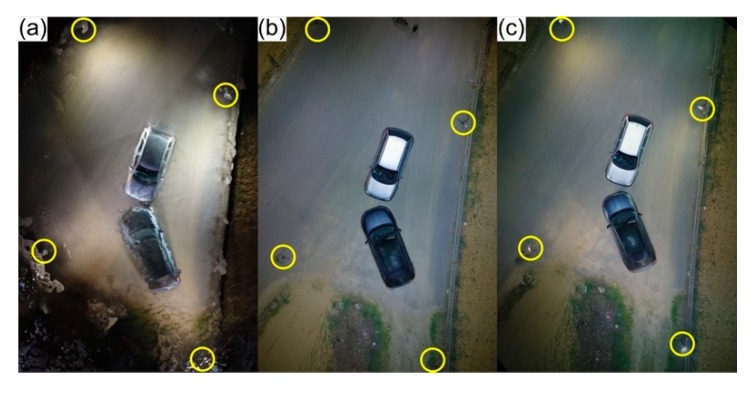
Orthophoto mosaics generated from aerial imagery acquired in an adverse light context: (**a**) only ground-based illumination; (**b**) portable illumination coupled to the unmanned aerial vehicle (UAV); and (**c**) combination of both ground and UAV-coupled illumination. Yellow circles mark the location of the four ground-based illumination units.

**Figure 12:**
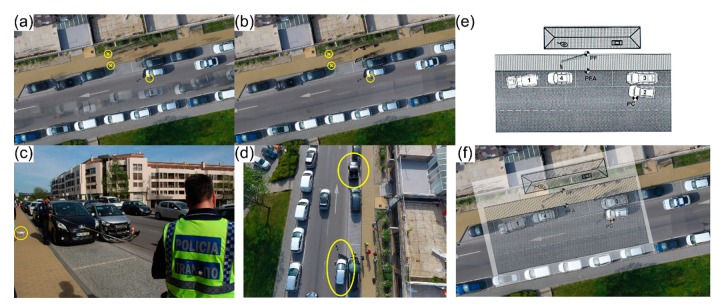
Proposed method applied to a road traffic accident in urban context: (**a**) orthophoto mosaic generated from the aerial imagery photogrammetric processing; (**b**) orthophoto mosaic without ghost-like elements; (**c**) Police officers taking measurements with a tape and the UAV getting ready to take-off; (**d**) aerial perspective of the scene, yellow ellipses identify the vehicles involved in the accident; (**e**) PSP’s sketch; and (**f**) overlap between PSP’s sketch and the generated orthophoto mosaic.

**Figure 13:**
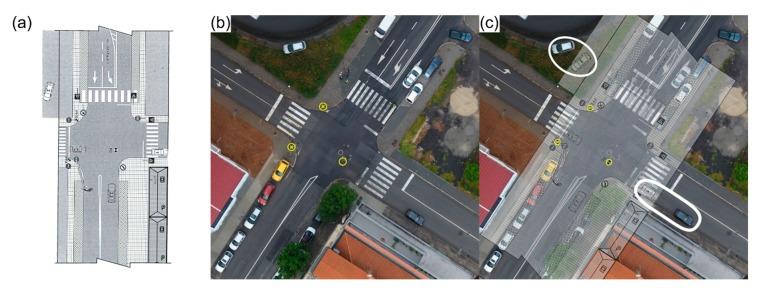
Sketch from Public Safety Police, based on data documented at the traffic accident scene with two vehicles involved (**a**). Orthophoto mosaic generated from the photogrammetric processing of aerial imagery (**b**), the three reference points considered in PSP’s sketch are highlighted using yellow circles: two traffic signs (yellow enclosed “×”) and the collision point (PC). Overlap between the accident sketch and the orthophoto mosaic, white ellipses mark the discrepancies regarding the position of both vehicles (**c**).

**Figure 14:**
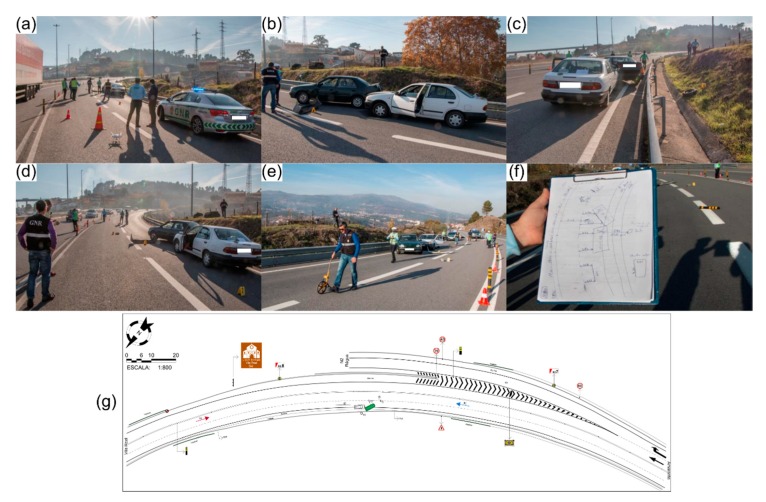
IP4 motorway road traffic accident scenario: different perspectives from the involved vehicles and debris (**a**, **b**, **c**, and **d**); National Republican Guard officers documenting the accident scene (**e**); the on-site handmade sketch (**f**); and scaled sketch produced by GNR in office (**g**).

**Figure 15:**
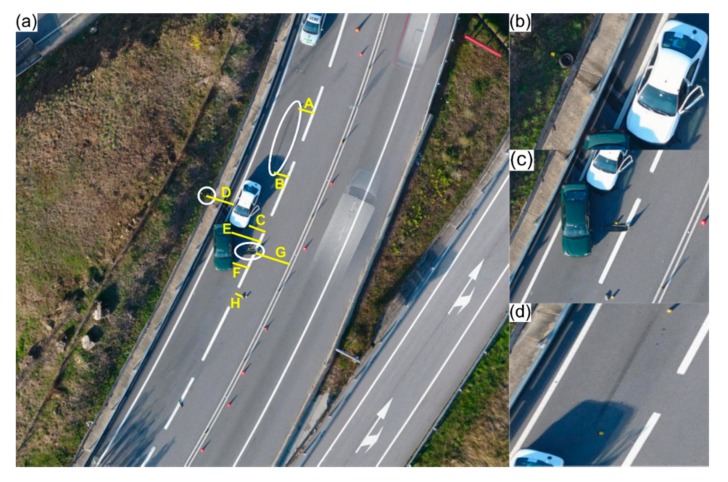
Part of the orthophoto mosaic of the IP4 motorway road traffic accident scenario (**a**). Both vehicles and other elements of interest (white ellipses): debris (**c**), skid marks (**d**), a tire (**b**). Measurement lines are also identified (A to H).

**Figure 16:**
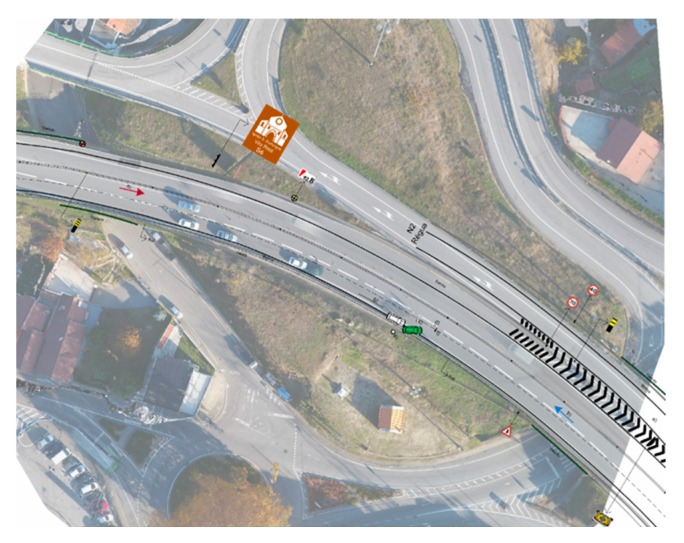
Overlap between National Republican Guard’s (GNR’s) sketch and the generated orthophoto mosaic.

**Figure 17:**
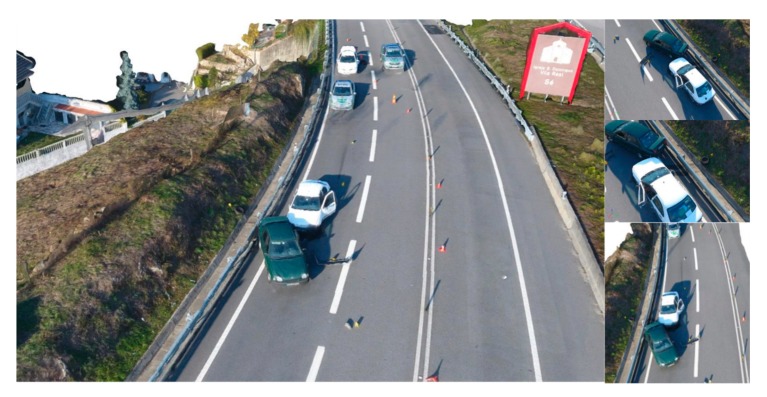
Different 3D perspectives from the road traffic accident scenario in a motorway (IP4).

The second simulation under adverse illumination conditions took place in a parking lot, where lighting was provided exclusively by public illumination fairly close to the area of interest. Regarding aerial imagery, four DG autonomous flights were carried out with (Figure 10b,d) and without (Figure 10a,c) the portable illumination coupled to the UAV: two at 20 m height (Figure 10a,b)—25 images were acquired in approximately 4 min and 30 s—and another two at 30 m height (Figure 10c,d)—17 images acquired in approximately 1 min and 40 s. All flights had a 70° camera angle and the portable illumination coupled to the UAV was manually adjusted to this angle. Lower flight heights were not considered due to the presence of trees and aerial cables in the scene surroundings.

All the presented outputs displayed quality reconstitutions of the scenario. Although public illumination was sufficient in this scenario, portable illumination coupled to UAVs can also be used as a complement, improving digital outputs light intensity/colour and compensating possible insufficient public illumination (e.g., poles that are too spaced out, missing or malfunctioning).

A third simulation under adverse illumination conditions was set in a suburban area, without public lighting support. Two vehicles—one of darker and one of brighter colour—were used and the accident scene was documented using: (i) only ground-based portable illumination (four units); (ii) only portable illumination coupled to the UAV; and (iii) a combination between ground-based and UAV-coupled illumination.

Regarding aerial imagery, several DG autonomous flights were carried out. Each flight had a 70° camera angle and the portable illumination was manually adjusted to this angle. The 25 m height flight—28 images acquired in approximately 5 min and 30 s—was considered for this scenario for two reasons: it is borderline regarding aerial imagery quality and this flight height allows for the presence of most type of obstacles. Figure 11 presents the final orthophoto mosaics focused on the vehicles involved in the simulation, for each aforementioned illumination setup.

The best visual result seems to be the one only using portable illumination coupled to the UAV (Figure 11b), as it seems to reduce shadow effects present in the combination between both portable illuminations (Figure 11c). Moreover, it also has simpler logistics for police forces when compared with ground-based illumination that requires setting up units in the crash scene.

## 6. Experimental Tests: Real Scenarios

This section presents some real road traffic accidents scenarios, addressed in cooperation with both PSP and GNR, in which the proposed methodology is compared with the current reconstitution method used by police forces.

### 6.1. Urban Context

Within the scope of a close collaboration with PSP of Vila Real, our research group accompanied PSP officers to some real road traffic accidents that happened within Vila Real urban context. Both teams worked roughly simultaneously to document crash scenes. Any road traffic accident scenario was disregarded if any of the involved parties did not concede authorization to disclose the acquired images. Only the most meaningful documented occurrences are presented. using their own approaches: the current reconstitution method and the proposed methodology, respectively. PSP sketches produced for the presented occurrences were provided for both comparison and validation purposes.

The first occurrence regards a daytime urban road traffic accident—one-way street, with two traffic lanes and parking in both sides of the street—that took place on 10 April 2017, directly involving two vehicles. PSP was called to the accident scene and proceeded by documenting it using its current methodology (Figure 12c). A sketch in Microsoft Visio (Microsoft Corporation, Redmond, USA) was produced afterwards using the data acquired.

As for the proposed methodology, a DG autonomous flight was carried out roughly at the same time that PSP officers were doing their investigation. The 20 m height flight—due to the presence of public illumination poles in the surroundings of the scene—50 images were acquired in approximately two minutes, with a 70° camera angle and 80% overlap between images. Figure 12a presents the orthophoto mosaic, reconstituting this road traffic accident scenario. Some debris and possible elements of interest can be clearly distinguished lying on the road. In the open traffic lane, vehicles passing by are depicted as ghost-like elements, but allow for comprehending traffic flow during the period of time lapsed between the road traffic accident and the end of PSP’s documenting process. Figure 12b presents the processed orthophoto mosaic without the ghost-like elements (e.g., vehicles and people on the sidewalk), allowing the viewer to focus on the crash scene elements only.

Figure 12f presents an overlap between PSP’s sketch (Figure 12e) and the processed orthophoto mosaic (Figure 12b), both in the same scale. There are some minor issues regarding the vehicles’ dimensions. However, considering that PSP officers use existing models from Microsoft Visio to develop their work, in this scenario both the sketch and the orthophoto mosaic are very similar. As a road traffic accident scenario reconstitution, the proposed approach provides a more complete set of outcomes, enabling different perspectives to be analysed (Figure 12d), zoom in a certain area, assess the overall accident scene environmental and physical contexts, precise location of debris and other elements of interest and capability to measure distances between any two points within the scene. Moreover, the accident scene is digitally preserved for analysis when necessary. This relatively simple urban road traffic accident only closed down one traffic lane for approximately 25 min (on-site documenting process), which meant that traffic could still flow in the other traffic lane. With the proposed approach, documenting the crash scene took only two minutes, with five more minutes spent in assembling the UAV and setting up the flight plan.

A second occurrence regards also a daytime urban road traffic accident, a busy intersection, that took place on May 11, 2017, involving two vehicles. PSP was also called to the traffic accident scene and proceeded by documenting it using its current methods. The produced sketch is presented in Figure 13a. Two traffic signs—a stop (PFA) and a wrong way (PFB) sign—and the collision point (CP) are considered as reference points. PSP officers took around 35 min to complete the on-site documenting process. As for the proposed methodology, a DG autonomous flight was carried out. The 20 m height flight—due to the presence of public illumination poles and buildings in the surroundings—acquired 57 images in approximately 2 min, with a 70° camera angle and 80% imagery overlap. Figure 13b presents the produced orthophoto mosaic of this road traffic accident scenario. The three reference points considered in PSP’s sketch (Figure 13a) are highlighted. Furthermore, debris and other possible elements of interest can be clearly distinguished lying on the road, near the collision point (PC).

Figure 13c presents an overlap between PSP’s sketch (Figure 13a) and the processed orthophoto mosaic (Figure 13b), both in the same scale. The main noticeable discrepancy regards the position of the vehicles. In a more complex road traffic accident scenario, the proposed approach would provide a more precise reconstitution, with a comprehensive context allowing for different perspectives, precise location of the debris, other elements of interest and the capability of measuring distances between any two points within the scene. Again, the crash scene is fully preserved for both present and future analysis. This urban road traffic accident scenario took approximately 35 min to be documented by PSP, with some impact in the usual traffic flow. With the proposed approach, documenting the entire crash scene took only 2 min, with 5 more minutes spent in assembling the UAV and setting up a proper flight plan.

### 6.2. Motorway Scenario

A motorway in the outskirts of Vila Real (IP4) is the last road traffic accident scenario, in real environment, presented in this work. It consisted on a simulated scene between two vehicles in an IP4 stretch with two lanes in one direction and one lane in another. The scenario was created to be particularly complex due to the overall physical context and to the scattered debris field, formed by objects of reduced dimensions (when compared to those of a vehicle), by skid marks and by an oil spill, put right next to one of the vehicles involved (different perspectives shown in Figure 14a–d). GNR was responsible both for staging the road traffic accident and by meticulously documenting it using its current method: a measuring wheel (Figure 14e), a measuring tape and a hand drawn sketch (Figure 14f). Officers took approximately 3 h to document the accident scene. The sketch was then used to produce two sketches in Microsoft Visio: one non-scaled—that took two hours to be done—and a scaled one (Figure 14g), that took 16 h to be completed.

As for the proposed methodology, a DG autonomous flight was carried out. The 25 m height flight encompassed a 67 × 40 m area, acquiring 135 images in approximately 5 min and 30 s, with a 70° camera angle and 80% imagery overlap. In only a few minutes, a substantial area—considering the crash scene dimensions—was quickly documented. Furthermore, the high-level of detail achieved can be seen in Figure 15. Debris scattered on the road, a car wheel in the ditch, skid marks and traffic cones circumscribing the accident scene are clearly identifiable.

Figure 16 presents an overlap between the scale sketch (Figure 14g) and the processed orthophoto mosaic (Figure 15a), both in the same scale. Notwithstanding the GNR officers experience and the painstaking documenting process carried out at the accident scene, there are some important discrepancies between the sketch and the orthophoto mosaic, independently on how they are aligned. Both images were put to the same scale and lined up considering motorway overpasses and the motorway guard rails next to the vehicles. Regarding the designated area of interest, debris positions are not correct, as are also the vehicles positions and skid marks. In a broader context, both the motorway profile and the respective reference elements do not match. Again, the proposed approach provides a more precise reconstitution, with a comprehensive context and debris precise location.

To further validate the proposed methods capability to preserve such a complex road traffic accident scenario for both present and future analysis, different perspectives of the traffic accident scene are shown in Figure 17 and that which determines the ability to measure distances between any two points within the scene. Based on GNR officers work in documenting the crash scene, Table 8 presents a comparison between on-site measurements and those obtained from the proposed approach. Moreover, each measured distance is represented in Figure 15a for clarification purposes. Considering tolerances, the maximum possible error is of 6 cm in measurement F (4% error), taken between the front of the green vehicle and the road line.

This motorway road traffic accident scenario took approximately 16 office hours to be represented in an official sketch. With the proposed approach, documenting the entire crash scene took approximately 5 min and 30 s (with 5 min more spent in assembling the UAV and setting up a proper flight plan) and approximately 30 min in the office to obtain the digital outputs. In addition to being faster, the proposed approach also enables precise measurements to be taken between two points in the crash scene, as well as different perspectives of the accident with a broader context. The digital outputs of the road traffic accident scenario reconstitution are made available for future analysis, if necessary.

## 7. Conclusions

Clearly, UASs allow for collecting more data with higher precision when compared to the current approaches for documenting road traffic accident scenes. However, in certain conditions, some field data retrieval may not be possible due to the presence of obstacles in the scenario—both in urban and rural contexts—such as trees, cables or buildings. Moreover, UAV-based photogrammetric methods can deal with adverse light conditions which remain scarcely addressed, leaving law enforcement authorities with no other option than to resort to the traditional approaches or others based on expensive and/or laboriously demanding technologies, whenever a road occurrence requiring their intervention takes place, for example, at night or on cloudy days. To tackle these issues, this study proposes and evaluates a low cost UAV-based photogrammetric methodology covering a wide range of scenarios, including: (i) obstacle-free scenes using only UAS as surveying instrument; (ii) scenes featured by road traffic accidents with partial inaccessibility for UAS usage, thus requiring complementary acquisition approaches; (iii) simulations in adverse light contexts (e.g., at night or with bad visibility), requiring the use of artificial light sources; and (iv) real road traffic accidents.

The several tests performed in this study and the literature review enabled us to obtain the benefits and limitations regarding the available approaches to address the digital reconstitution road traffic accident scenes, which are presented in Table 9.

Despite the number of valid traffic accident research works involving the use of UAV-based surveying, most of them were developed considering ideal scenarios, either on urban or rural environments. To address this gap, a methodology consisting of a full pipeline to digitally document road traffic accidents using UAVs, ground cameras, artificial light sources and photogrammetry was proposed in this technical note. A preliminary set of experiments were carried out to determine default flight parameters on ideal scenarios, which can be fine-tuned according with each set of conditions characterising a road traffic accident occurrence. Afterwards, to demonstrate the usefulness of UAVs on traffic accidents investigation, a set of experiments was performed, more specifically: (i) considering areas populated with typical obstacles found in urban and rural settings, at different densities; and (ii) under unfavourable illumination conditions. Furthermore, practical situations were addressed with the Portuguese road traffic authorities, wherein traditional approach and proposed methodology are compared in terms of efficiency and accuracy.

Encouraging results were obtained from the assessment of the different experiments, which attest the feasibility of using UAVs for traffic accident investigation in many environments characterised by several conditions. Regarding extreme situations wherein traffic accident scenarios are under severe influence of obstructing obstacles, other alternative/complementary strategies to digitally document evidences can be used at the surveying stage, by using, for example consumer-grade digital cameras or smartphones. Moreover, in regards to the tests carried out in real situations, in simpler scenarios the results of both traditional approaches and the proposed methodology reached a fair level of similarity. However, in more complex cases, significant advantages of the latter were observed in relation to the former in terms of accuracy and operation times.

The complete solution implementing the proposed methodology requires the integration of the following components: (1) aerial platform (UAS), endowed with artificial lighting to operate in the most varied illumination conditions; (2) several types of sensors, according to the specificity of the operation and; (3) a computer application—freely available or developed from scratch—to generate the required digital documentation products, for consistent and non-redundant storing, enabling a quick assessment and retrieval of reliable measurements. Forthcoming directions include: motivating the change on actual traffic road accidents documentation paradigm with promotion and training initiatives—that have already started to take place with local authorities—aiming to complement and, eventually, replace the traditional approach with a technological pipeline, pursuing not only a normalised digital documentation process but also the reduction of required times and endeavour in current practices carried out in the field for surveying purposes. Another important issue to address in future is the need to have UAVs operating in a wider number of nonextreme—though unfavourable—weather situations, in which structural upgrades such as UAV shield adjustments for waterproofing and hardware improvements for fast compensation of windy conditions may be expected, eventually, under the manufacturer’s initiative. The improvement of the methodology’s pipeline by implementing features for parallel processing while the UAV is surveying road traffic accidents, suppressing the need for post-processing activities in the office should also be addressed. Real-time online access to reconstructed road traffic accident scenes is another relevant capability to be developed. Moreover, layers of computational intelligence and analytics are being designed to enhance the proposed methodology with decision support features that may assist authorities, urban planning professionals and municipal entities in the continuous improvement of the road traffic system.

## Figures and Tables

**Table 1:** Foreseen proposed methodology road traffic accident scenarios and respective imagery acquisition processes. UAV: unmanned aerial vehicle.

Foreseen Road Traffic Accident Scenarios	Imagery Acquisition Process
Absence of obstacles (ideal situation)	A UAV can carry out a fully autonomous flight, planned with proper software
Presence of some obstacles, restricting UAV access to (at least) one perspective	Image acquisition can be complemented either using manual flight (for wider areas) or by using a ground camera, such as a smartphone (for confined areas of a few squared meters, orbital style acquisition)
Presence of obstacles that completely inhibit the use of UAVs	Image acquisition is done only by using a ground camera, such as a smartphone (more suitable for confined areas of a few squared meters, orbital style acquisition)
Adverse light conditions	Artificial light sources can be setup around the area of interest. As an alternative/complement, whenever the use of UAVs is possible, portable lightweight light sources can be mounted on the UAV, ensuring the camera is neither occluded or overexposed.

**Table 2:** Most representative combinations of flight parameters and photogrammetric processing duration (PPD), considering the best possible compromise between flight height and camera angle. Flight typology as simple grid (SG) or double grid (DG); GSD: ground sampling distance.

Height (m)	Camera Angle	Flight Typology	Number of Images	Flight Duration (min:s)	GSD (cm)	PPD (min:s)
40	90	SG	18	3:52	1.70	3:32
40	80	DG	15	2:27	1.54	4:33
40	65	DG	36	3:39	1.81	9:25
30	90	SG	18	3:35	1.21	3:03
30	80	DG	36	3:27	1.08	11:11
30	65	DG	36	3:27	1.33	7:43
15	90	SG	32	4:34	0.65	4:36
15	80	DG	72	4:58	0.59	21:59
15	65	DG	76	4:56	0.72	14:02
10	30–60	Manual	76	4:46	0.34	30:18

**Table 3:** Comparison between the reference measurements and those most representative computed from photogrammetric-based virtual models obtained from flight missions. Combinations of different flight parameters, such as flight mode—manual or autonomous (single/double grid)—flight height and camera angle are presented for each flight mission. The most accurate result is highlighted in bold.

Measurement	Distance (cm)			
	AB	CE	DE	AC
Measuring tape	286	165	93	249
40 m SG (90°)	292 ± 2	169 ± 1	95 ± 2	255 ± 1
30 m SG (90°)	288 ± 2	166 ± 2	94 ± 2	250 ± 1
15 m SG (90°)	286 ± 1	165 ± 1	93 ± 1	248 ± 1
40 m DG (65°)	289 ± 2	166 ± 1	94 ± 1	250 ± 2
30 m DG (65°)	284 ± 1	165 ± 2	93 ± 1	247 ± 1
**15m DG (65°)**	**286 ± 1**	**165 ± 1**	**93 ± 1**	**249 ± 1**
40 m DG (80°)	285 ± 2	164 ± 2	92 ± 1	247 ± 3
30 m DG (80°)	283 ± 1	163 ± 1	92 ± 1	246 ± 1
15 m DG (80°)	286 ± 1	164 ± 1	93 ± 1	249 ± 1
Manual	287 ± 1	166 ± 1	94 ± 1	251 ± 1

**Table 4:** Comparison between measurements taken using a measuring tape, the proposed UAV-based photogrammetric approach, a total station and a GNSS receiver. The difference to the reference measurements (measuring tape) to the one taken by the other equipment is also provided.

Distance	Tape (cm)	GNSS (cm)	Total station (cm)	UAV (cm)
AB	257	259 (−2)	258 (−1)	258 (−1)
BC	180	179 (1)	180 (0)	181 (−1)
CD	268	267 (1)	269 (−1)	269 (−1)
EF	264	262 (2)	264 (0)	265 (−1)
GH	264	263 (1)	264 (0)	264 (0)
AH	377	380 (−3)	377 (0)	378 (−1)
BG	440	443 (−3)	440 (0)	441 (−1)
CF	368	370 (−2)	369 (−1)	369 (−1)
DE	375	377 (−2)	376 (−1)	376 (−1)

**Table 5:** Comparison between ground-truth measurements and the UAV-based photogrammetric approach relative to road traffic accident in a context with poles and aerial cables. Errors associated with the UAV-based measurements were calculated using Pix4D.

Distance	Tape (cm)	Point Cloud (cm)
AB	451	453 ± 4
CD	117	117 ± 1
EF	42	42 ± 1

**Table 6:** Comparison between ground-truth measurements taken and the measurements obtained from the UAV-based point cloud in a simulated road traffic accident where trees obstructs part the involved vehicles. Errors associated with the UAV-based measurements were calculated using Pix4D.

Distance	Tape (cm)	Point Cloud (cm)
AB	69	69 ± 2
BD	86	87 ± 1
CD	77	78 ± 2
AC	122	123 ± 2
EF	79	80 ±1

**Table 7:** Comparison between the ground-truth measurements with those obtained from the generated point cloud where a building is partially obstructing a portion of a vehicle.

Distance	Tape (cm)	Point Cloud (cm)
AB	459	459 ± 1
BC	251	251 ± 1
DE	268	269 ± 1
DF	669	669 ± 1

**Table 8:** Comparison between measurements taken by National Republican Guard (GNR) officers with those obtained from the digital products generated by the proposed approach, for the motorway (IP4) road traffic accident. Errors associated with the proposed methodology measurements were calculated using Pix4D.

Distance Line	Tape (cm)	Point Cloud (cm)
A	163	166 ± 1
B	163	163 ± 3
C	163	162 ± 3
D	260	260 ± 2
E	252	254 ± 4
F	143	137 ± 4
G	300	300 ± 2
H	70	71 ± 1

**Table 9:** Comparison between the available approaches to address road traffic accident scenes reconstitution: pros, cons and possible outputs.

Approach	Pros	Cons	Outputs
Measuring Tape or Wheel	✓Ease-of-use ✓Low cost	✕Time-consuming ✕Prone to errors ✕No visual data ✕Data not georeferenced	-Manual measurements
Sketches & Photographs	✓Ease-of-use ✓Low cost	✕Time-consuming ✕Inaccurate ✕Data not georeferenced	-Measurements -Images -Forensic sketches
Total Station	✓Rigorous	✕Time-consuming ✕Skilled operator ✕Not suitable for mobility ✕Considers line-of-sight ✕Expensive	-Precise measurements (angles and distances)
Laser Scanning	✓Rigorous ✓3D Visualisation	✕Time-consuming ✕Loss of information due to its position ✕Confined action range ✕Expensive	-Point clouds -Images
GNSS Receivers	✓Rigorous	✕Time-consuming ✕Prone to signal obstruction ✕Expensive	-Precise georeferenced points
UAS	✓Ease-of-use ✓Large coverage ✓Multiple outputs ✓3D Visualisation ✓Cost-effective	✕Data acquisition constrains ✕Liable to collisions ✕Requires training ✕Rigidly Regulated	-Point clouds -3D meshes -Orthophoto mosaics -Digital Surface Models
